# La _1−*x*_*Ca*_*x*_*MnO*_3_ semiconducting nanostructures: morphology and thermoelectric properties

**DOI:** 10.1186/1556-276X-9-415

**Published:** 2014-08-21

**Authors:** Mario Culebras, Raquel Torán, Clara M Gómez, Andrés Cantarero

**Affiliations:** 1 Materials Science Institute, University of Valencia, P. O. Box 22085, Paterna, Valencia, Spain

**Keywords:** Nanostructures, Seebeck, Thermoelectricity, Perovskites

## Abstract

Semiconducting metallic oxides, especially perosvkite materials, are great candidates for thermoelectric applications due to several advantages over traditionally metallic alloys such as low production costs and high chemical stability at high temperatures. Nanostructuration can be the key to develop highly efficient thermoelectric materials. In this work, La _1−*x*_*Ca*_*x*_*MnO*_3_ perosvkite nanostructures with Ca as a dopant have been synthesized by the hydrothermal method to be used in thermoelectric applications at room temperature. Several heat treatments have been made in all samples, leading to a change in their morphology and thermoelectric properties. The best thermoelectric efficiency has been obtained for a Ca content of *x*=0.5. The electrical conductivity and Seebeck coefficient are strongly related to the calcium content.

## Background

Over the last decade, there has been an increasing interest in finding new highly efficient thermoelectric materials for electronic cooling [1-3] and power generation [4-6]. The energy demand in developed and under-developed countries is increasing due to the population growth and the improvement of the standard level of life in emerging countries. Unfortunately, reserves of fossil fuels are not unlimited, and their use generates huge amounts of CO _2_ in the atmosphere. Many human activities (power plants, cement plants, steel mills, and vehicles engines as a few examples) are generating high amount of waste heat at different ranges of temperature. The conversion of this waste heat into electric energy would be an important contribution to the sustainable development as it would allow to reduce both the Greenhouse gas emissions and fossil fuel consumption. Thermoelectric generators are designed to convert a temperature difference into electricity (Seebeck effect) or, inversely, electric energy into a thermal gradient (Peltier effect). Thermoelectric materials must have a high conversion efficiency, and they must also be composed conveniently of non-toxic and abundantly available elemental species having high chemical stability in air. The performance of a thermoelectric material is determined by the dimensionless figure of merit ZT:

(1) $$\text{ZT}=\frac{\sigma S^{2}}{\kappa }T\;,$$

*S* being the Seebeck coefficient, *σ* the electrical conductivity, *κ* the thermal conductivity, and *T* the absolute temperature. The power factor (PF) defined as PF≡*σS*^2^ can be used to compare the relative efficiency when the thermal conductivity is similar in different samples.

Over the past 30 years, semiconductor alloys based on Bi _2_*Te*_3_, PbTe, and SiGe [7-9] have been extensively studied and optimized for their use in thermoelectric applications. However, most of these compounds present disadvantages related to the shortage of raw materials, toxicity, or high costs of production.

For these reasons, research on the new materials to build up efficient thermoelectric devices is a scientific subject of current interest [10,11]. Recently, several oxides such as NaCoO _2_[12], Ca _3_*Co*_4_*O*_9_[13], Sr _1−*x*_*La*_*x*_*TiO*_3_[14], La _1−*x*_*Sr*_*x*_*CoO*_3_[15], Nd _1−*x*_*Ca*_*x*_*CoO*_3_[16], or Ca _0.8_*Dy*_0.2_*MnO*_3_[17] have shown excellent thermoelectric properties. More precisely, perosvkite-type transition metal oxide single crystals have depicted large thermoelectric responses [14]. The electrical properties of La _1−*x*_*A*_*x*_*MnO*_3_ (A = Ca, Sr, Ba, and Pb) perosvkite-type oxides are related to their stoichiometry [14]. Significant variations appear when the degree of substitution of the alkali-earth element for La varies from 0% to 50% [14]. The novelty of perovskite-type oxides is due to their low cost, non-toxicity, and possibility of being used for high-temperature applications. The origin of the thermoelectric properties in these oxides is not yet fully understood, but it could be related to the high spin-orbit interaction as well as the large electron effective mass [14].

In 1993, the work of Hicks and Dresselhaus [18] suggested that the morphology of a thermoelectric system can be used to improve both the electronic transport and the phonon scattering. Nanostructuration can increase ZT over unity by changing *σ* and *S* independently. The density of electronic states in a nanostructured system, when the Fermi energy is close to a maximum in the density of electronic states, depicts usually sharp peaks and theoretically larger Seebeck coefficients than the same material in bulk [19]. Furthermore, the phonon dynamics and heat transport in a nanostructured system can be suppressed by means of size effects. Nanostructures with one or more dimensions smaller than the phonon mean free path (a phonon glass) but larger than that of electrons (electron crystal) will noticeably reduce the thermal conductivity *κ* without affecting much the electrical transport. In other words, phonon transport will be strongly disturbed, while the electronic transport can remain bulk-like in nanostructured systems.

In this report, La _1−*x*_*Ca*_*x*_*MnO*_3_ nanocrystals have been obtained by the hydrothermal method as a function of the Ca content. Several heat treatments have been made to determine the temperature when the perosvkite phase is obtained. Scanning electron microscopy and X-ray diffraction studies have been used to determine the perosvkite phase. The electrical conductivity and Seebeck coefficient have been determined as a function of temperature in order to analyze their thermoelectric performance.

## Methods

### Materials

The reactants MnCl _2_·4H _2_O, Ca(NO _3_) _2_, La(NO _3_) _3_, KMnO _4_ and KOH were purchased from Sigma Aldrich Co., Madrid, Spain.

### Synthesis of La _1−*x*_*Ca*_*x*_*MnO*_3_ nanostructures

La _1−*x*_*Ca*_*x*_*MnO*_3_ samples with *x*=0.005,0.05,0.1 and 0.5 have been prepared by a conventional hydrothermal treatment [20-22]. Stoichiometric amounts of reactants were used to have an aqueous solution of 0.55 M in cations (Mn ^7+^, Mn ^2+^, Ca ^2+^, and La ^3+^) by keeping a molar ratio between KMnO _4_ and MnCl _2_·4H _2_O according to the average valence of Mn ions in La _1−*x*_*Ca*_*x*_*MnO*_3_. The pH of the solution was adjusted to 13 by adding KOH. After ultrasonic stirring, the solution was transferred into a Teflon autoclave and heated for 30 h at 230°C. Then, the reactor was cooled down to room temperature, and the obtained solid was washed with water and ethanol and dried at 230°C for 12 h. The powder was subjected to different temperatures, 650°C and 900°C for 12 h. The powder obtained after 900°C was pressed to form compact pellets (0.5-in. diameter) by using a pellet die at 490 MPa. Further, the pellet was sintered at 900°C for 24 h.

### Characterization

The scanning electron microscopy (SEM) analysis was carried on a Hitachi 4800S microscope (Hitachi, Ltd., Tokyo, Japan) at an acceleration voltage of 20 kV and at a working distance of 14 mm for gold-coated surfaces. The wide-angle X-ray diffraction (WAXRD) patterns were acquired on a Bruker AXS D5005 diffractometer (Bruker AXS GmbH, Karlsruhe, Germany). The samples were scanned at 4°/min using Cu K _*α*_ radiation (*λ*=0.15418 nm) at a filament voltage of 40 kV and a current of 20 mA. The diffraction scans were collected within the 2*θ*= 20° to 80° range with a 2*θ* step of 0.01°.

The electrical conductivity has been determined by means of the van der Pauw method [23,24], where four contacts are used to eliminate the effect of the contact resistance. The electrical conductivity can be obtained from two four-point resistance measurements independently either on contact resistances or on the specific geometry of the contact arrangement. For the first resistance measurement, a current *I*_*AC*_ is driven from two contacts, named *A* and *C*, and the potential difference *V*_*BD*_ between the other two contacts, *B* and *D*, was measured, giving the first resistance *R*_1_=*V*_*BD*_/*I*_*AC*_. The second resistance, *R*_2_=*V*_*AB*_/*I*_*CD*_, is obtained by driving the current from *C* to *D* and measuring the voltage between *A* and *B*. The conductivity of the sample is obtained by solving the van der Pauw equation:

(2) $$e^{-\mathrm{\pi d}R_{1}\sigma }+e^{-\mathrm{\pi d}R_{2}\sigma }=1\;,$$

where *d* is the sample thickness. A Keithley 2400 current source (Keithley Instruments Inc., Cleveland, OH, USA) was used as driving source.

The Seebeck coefficient has been measured with a homemade apparatus. In order to control the temperature, we used a Lakeshore 340 temperature controller, and to record the potential data, a Keithley 2750 Multimeter/Switching System was used. The Seebeck coefficient can be determined as the ratio between the electrical potential, *ΔV*, and the temperature difference, *ΔT*, that is,

(3) $$S=\frac{\mathrm{\Delta V}}{\mathrm{\Delta T}}\;.$$

## Results and discussion

**Figure 1:**
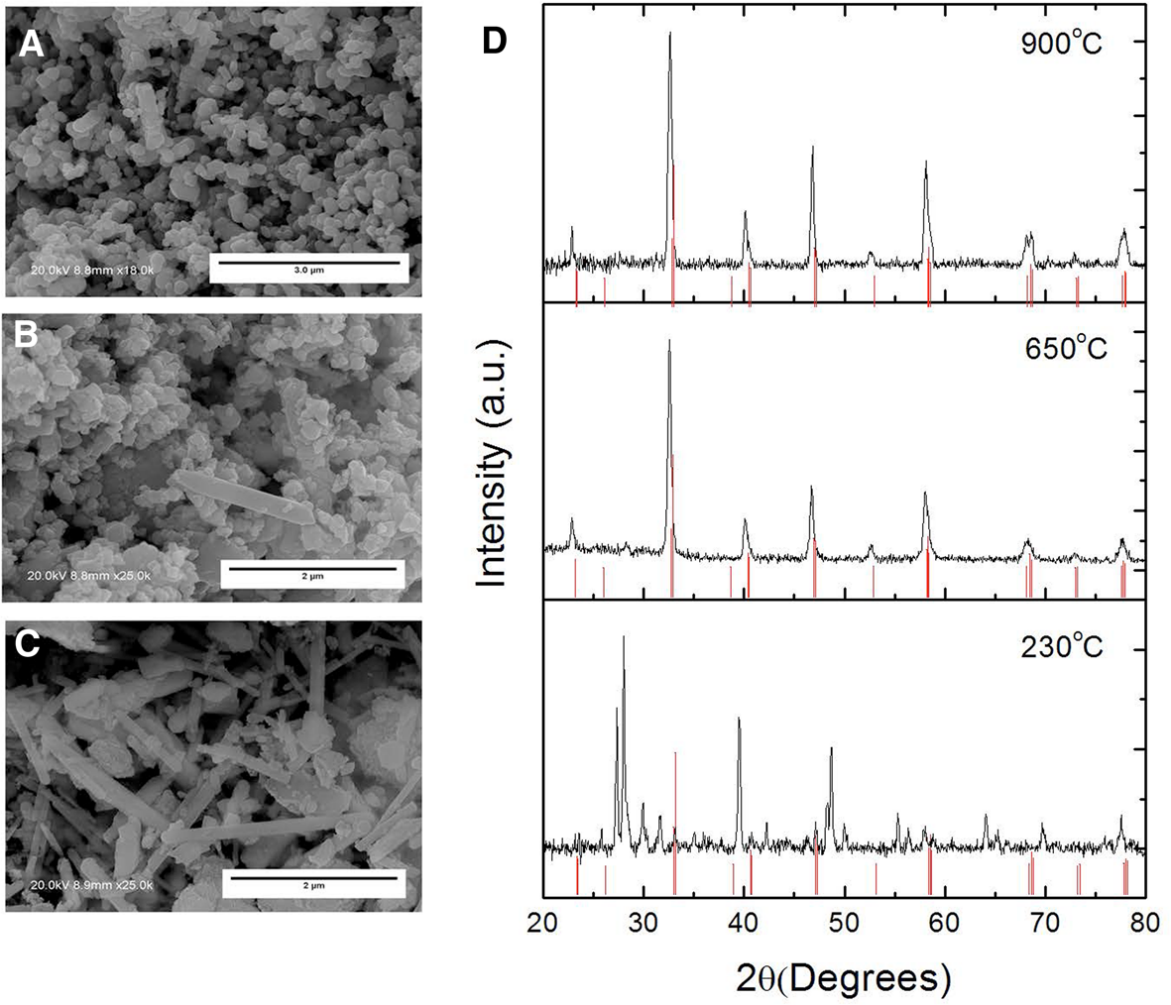
**Scanning electron microscopy images after different temperature treatments for 12 h.(A)** 900°C, **(B)** 650°C, and **(C)** 230°C. **(D)** X-ray diffraction spectra of La _1−*x*_*Ca*_*x*_*MnO*_3_ nanostructures (*x*=0.05). The red lines refer to the perosvkite phase diffraction pattern.

Scanning electron microscopy images show the evolution of the morphology as a function of temperature treatment (Figure 1A,B,C). The first temperature treatment was carried out at 230°C for 12 h (drying treatment); the resultant morphology after this treatment is shown in Figure 1C. A fibrillar morphology has been observed after this treatment, with an average diameter of 120±50 nm. The second treatment was carried out at 650°C for 12 h, leading to a change in the morphology, from fibrillar to aggregated nanoparticles as shown in Figure 1B, although some parts of the powder retained the fibrillar morphology. Finally, the last treatment was carried out at 900°C for 12 h, as shown in Figure 1A; all the material depicts a nanoparticle structure. This evolution of the morphology with temperature is similar to that observed in others materials like La _1−*x*_*Sr*_*x*_*CoO*_3_, previously reported in the literature [25].

The X-ray diffraction patterns for the La _1−*x*_*Ca*_*x*_*MnO*_3_ (*x*=0.05) powder, resulting from the thermal treatment at 230°C, 650°C, and 900°C are depicted in Figure 1D. Similar diffraction patterns are obtained for all the samples regardless the Ca content. X-ray diffraction analysis has been made in order to know when the orthorhombic perovskite phase appears because only this phase presents thermoelectric activity [26-28]. At 230°C, the perovskite phase was not obtained, resulting in an insulating material. The diffraction peaks observed at 230°C are related to segregated metallic oxides of Ca, La, and Mn (CaO, Mn _3_*O*_4_, CaMn _2_*O*_4_, etc.). At 650°C, the WAXDR spectrum indicates that the orthorhombic perovskite-type structure was present. The material obtained after this treatment was a semiconductor material. The WAXDR spectrum of the sample heated at 900°C is similar to that obtained at 650°C, indicating that most of the material has the perovskite phase. The perosvkite phase is attained at 650°C; however, the electrical conductivity of the compacted powder (without sintering) obtained at 650°C and 900°C is very low (around 10 ^−3^ S/cm). In addition, the sample size and shape are more homogeneous after treatment at 900°C. Thus, in order to use these materials for thermoelectric applications, we have realized a sintering process by keeping the compact pellet at 900°C for 24 h.

**Figure 2:**
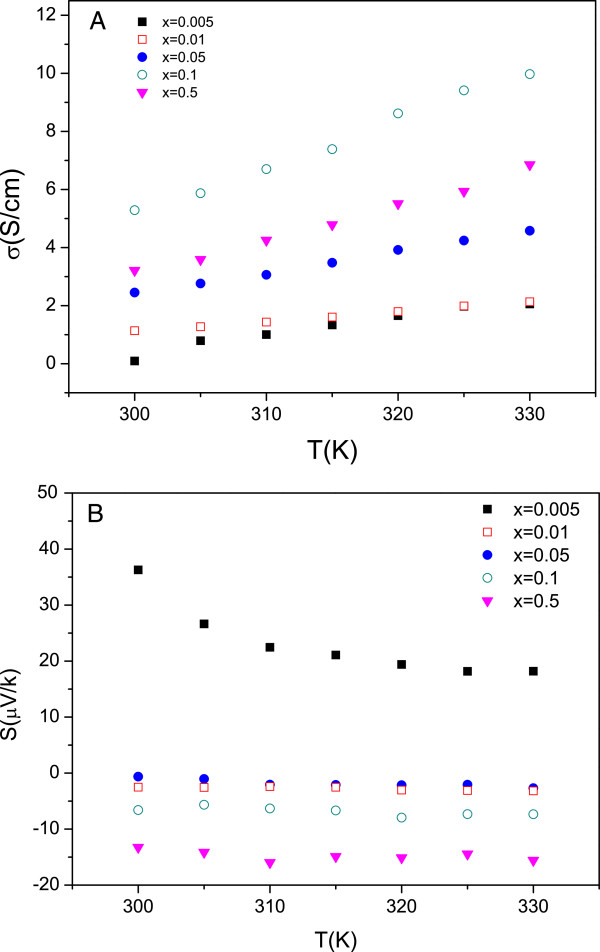
**Electrical conductivity and Seebeck coefficient.(A)** Electrical conductivity and **(B)** Seebeck coefficient of La _1−*x*_*Ca*_*x*_*MnO*_3_ after the sintering process as a function of temperature.

The electrical conductivity of the samples after the sintering process is plotted in Figure 2A. An increase of 3 orders of magnitude with respect to the samples before the sintering process is observed. This fact can be explained by the reduction of the interfaces and grain boundaries during the sintering process. The electrical conductivity increases with temperature; this trend is expected in semiconducting materials [29,30]. The maximum value of the electrical conductivity, 10 S/cm, has been obtained for La _0.9_*Ca*_0.1_*MnO*_3_ at 330 K. The increase of the calcium content in the nanostructured material produces an enhancement of the electrical conductivity, with the exception of La _0.5_*Ca*_0.5_*MnO*_3_. Figure 2B shows the variation of the Seebeck coefficient with the temperature and Ca content. The values of *S* change from positive to negative at high Ca content, denoting a change from p-type to n-type conduction. The dependence of *S* with temperature is negligible except for the lower Ca content (*x*=0.005).

Generally, a p-type conductivity is observed in LaMnO _3_[31,32]. It has been attributed to the excess of oxygen (O _3+*δ*_) and La vacancies and probably also to Mn vacancies [33], although it is not completely clear. Doing a literature search, it is clear that LaMnO _3_ is a p-type semiconductor, while CaMnO _3_ is an n-type semiconductor and contains an oxygen defect (O _3−*δ*_). In the work of Zeng et al. [34], electrical conductivity is analyzed as a function of the oxygen defect and they obtain a decrease of the activation energy as soon as the defect of oxygen is higher. From these observations, we can argue that the type of conduction in La _1−*x*_*Ca*_*x*_*O*_3_ goes from p to n as soon as the Ca content increases. We have found in our measurements that only the sample with *x*=0.005 is a p-type semiconductor, while all the samples with a higher Ca concentration are n-type semiconductors. There are several empirical models in the literature [27,33] to explain the conductivity based on different vacancies, but the location of the Mn(d) and O(p) levels is not clear. There are also several *ab initio* calculations, but we have found contradictions in the location of the Mn(d) and O(p) levels, probably due to the Jan-Teller distortion.

The power factor has been calculated in order to estimate the thermoelectric efficiency in this kind of materials at 330 K (Table 1). The best power factor, 0.16 *μ*W m ^−1^ K ^−2^ has been reached in the La _0.5_*Ca*_0.5_*MnO*_3_ sample. The values estimated in this work are similar to those found in organic semiconductors [35-37].

**Table 1:** **Thermoelectric parameters of La **_1−***x***_ ***Ca***_***x***_ ***MnO***_**3**_** nanostructures at 330 K**

**Sample**	***σ* (S/cm)**	***S* ( *μ *** **V/K)**	**Power factor ( *μ *** **W/mK**^**2**^**)**
La _0.995_*Ca*_0.005_*MnO*_3_	2.05	18.18	0.068
La _0.99_*Ca*_0.01_*MnO*_3_	2.13	−2.69	0.002
La _0.95_*Ca*_0.05_*MnO*_3_	4.57	−3.18	0.003
La _0.9_*Ca*_0.1_*MnO*_3_	10.00	−7.35	0.053
La _0.5_*Ca*_0.5_*MnO*_3_	6.85	−15.577	0.166

## Conclusions

La _1−*x*_*Ca*_*x*_*MnO*_3_ perovskite nanostructures have been synthesized by the hydrothermal method. The perovskite-type structure has been obtained at 650°C and 900°C. The nanostructure morphology changes from fibrillar to nanoparticle type when increasing the temperature treatment. The electrical conductivity increases 3 orders of magnitude after the sintering process. The electrical conductivity depends on the calcium content. The sign of Seebeck coefficient changes from positive to negative. The best power factor of 0.16 *μ*V/mK ^2^ has been obtained for the sample La _0.5_*Ca*_0.5_*MnO*_3_. The magnitude of PF indicates that these materials have a modest efficiency at room temperature. More research is needed in order to increases the thermoelectric efficiency.

## Competing interests

The authors declare that they have no competing interests.

## Authors’ contributions

MC was in charge of the thermoelectric characterization, RT developed the synthesis of materials, CMG was in charge of X-ray analysis, and AC realized the discussion of the thermoelectric results. All authors read and approved the final manuscript.

## References

[B1] Kim M-Y Oh T-S **Thermoelectric power generation characteristics of a thin-film device consisting of electrodeposited n-Bi**_ **2** _**Te**_ **3** _** and p-Sb**_ **2** _**Te**_ **3** _** thin-film legs** J Electron Mater 2013 9 9 2752 2757 10.1007/s11664-013-2671-3

[B2] Zhao D Tan G **A review of thermoelectric cooling: materials, modeling and applications** Appl Therm Eng 2014 9 1–2 15 24

[B3] Sharma S Dwivedi VK Pandit SN **Exergy analysis of single-stage and multi stage thermoelectric cooler** Int J Energy Res 2014 9 2 213 222 10.1002/er.3043

[B4] Yoon CK Chitnis G Ziaie B **Impact-triggered thermoelectric power generator using phase change material as a heat source** J Micromech Microeng 2013 9 11 114004 10.1088/0960-1317/23/11/114004

[B5] Jo S-E Kim M-S Kim M-K Kim Y-J **Power generation of a thermoelectric generator with phase change materials** Smart Mater Struct 2013 9 11 115008 10.1088/0964-1726/22/11/115008

[B6] Hourdakis E Nassiopoulou AG **A thermoelectric generator using porous Si thermal isolation** Sensors 2013 9 10 13596 13608 10.3390/s131013596 24152923 PMC3859081

[B7] Saleemi M Toprak MS Li S Johnsson M Muhammed M **Synthesis, processing, and thermoelectric properties of bulk nanostructured bismuth telluride (Bi**_ **2** _**Te**_ **3** _**)** J Mater Chem 2012 9 2 725 730 10.1039/c1jm13880d

[B8] Semizorov A **A study of pressed thermoelectric-materials based on Bi**_ **2** _**Te**_ **3** _**-Sb**_ **2** _**Te**_ **3** _**-Sb**_ **2** _**Se**_ **3** _** solid-solutions** Inorg Mater 1995 9 6 675 677

[B9] Hasapis TC Girard SN Hatzikraniotis E Paraskevopoulos KM Kanatzidis MG **On the study of PbTe-based nanocomposite thermoelectric materials** J Nano Res 2012 9 165 174

[B10] Ghrairi N Bouaicha M **Structural, morphological, and optical properties of TiO**_ **2** _** thin films synthesized by the electrophoretic deposition technique** Nanoscale Res Lett 2012 9 357 10.1186/1556-276X-7-357 22747886 PMC3458948

[B11] Mula G Manca L Setzu S Pezzella A **Photovoltaic properties of PSi impregnated with eumelanin** Nanoscale Res Lett 2012 9 1 1 9 10.1186/1556-276X-7-1 22776626 PMC3420258

[B12] Terasaki I Sasago Y Uchinokura K **Large thermoelectric power in NaCo**_ **2** _**O**_ **4** _** single crystals** Phys Rev B 1997 9 12685 12687 10.1103/PhysRevB.56.R12685

[B13] Masset A Michel C Maignan A Hervieu M Toulemonde O Studer F Raveau B Hejtmanek J **Misfit-layered cobaltite with an anisotropic giant magnetoresistance: Ca**_ **3** _**Co**_ **4** _**O**_ **9** _Phys Rev B 2000 9 1 166 175 10.1103/PhysRevB.62.166

[B14] Okuda T Nakanishi K Miyasaka S Tokura Y **Large thermoelectric response of metallic perovskites: Sr **_1***−******x***_**La**_ ** *x* ** _**TiO**_ **3** _** (0**** *<* **** *x* **** *<* ****0.1)** Phys Rev B 2001 9 113104

[B15] Berggold K Kriener M Zobel C Reichl A Reuther M Müller R Freimuth A Lorenz T **Thermal conductivity, thermopower, and figure of merit of La **_1***−******x***_**Sr**_ ** *x* ** _**Co**_ **3** _Phys Rev B 2005 9 155116

[B16] Culebras M Gomez C Gomez A Sapina F Cantarero A **Synthesis of Nd **_1***−******x***_**Ca**_ ** *x* ** _**CoO**_ **3** _** perovskite nanowires for thermoelectric applications** J Elect Eng 9 59 64

[B17] Park K Lee GW **Thermoelectric properties of Ca**_ **0.8** _**Dy**_ **0.2** _**MnO**_ **3** _** synthesized by solution combustion process** Nanoscale Res Lett 2011 9 548 10.1186/1556-276X-6-548 21974984 PMC3212086

[B18] Hicks LD Dresselhaus MS **Effect of quantum-well structures on the thermoelectric figure of merit** Phys Rev B 1993 9 19 12727 12731 10.1103/PhysRevB.47.12727 10005469

[B19] Humphrey TE Linke H **Reversible thermoelectric nanomaterials** Phys Rev Lett 2005 9 096601 15783983 10.1103/PhysRevLett.94.096601

[B20] Wang Y Fan HJ **Improved thermoelectric properties of La **_1***−******x***_**Sr**_ ** *x* ** _**CoO**_ **3** _** nanowires** J Phys Chem C 2010 9 32 13947 13953 10.1021/jp105367r

[B21] Zhang T Jin C Qian T Lu X Bai J Li X **Hydrothermal synthesis of single-crystalline La**_ **0.5** _**Ca**_ **0.5** _**MnO**_ **3** _** nanowires at low temperature** J Mater Chem 2004 9 18 2787 2789 10.1039/b405288a

[B22] Zhu X Wang J Zhang Z Zhu J Zhou S Liu Z Ming N **Perovskite nanoparticles and nanowires: microwave-hydrothermal synthesis and structural characterization by high-resolution transmission electron microscopy** J Am Ceram Soc 2008 9 8 2683 2689 10.1111/j.1551-2916.2008.02494.x

[B23] Van Der Pauw LJ **A method of measuring the resistivity and Hall coefficient on lamellae of arbitrary shape** Philips Tech Rev 1958 9 220 224

[B24] de Boor J Schmidt V **Complete characterization of thermoelectric materials by a combined van der Pauw approach** Adv Mater 2010 9 38 4303 4307 10.1002/adma.201001654 20626012

[B25] Deng J Zhang L Dai H He H Au CT **Single-crystalline La**_ **0.6** _**Sr**_ **0.4** _**CoO **_3***−δ***_** nanowires/nanorods derived hydrothermally without the use of a template: catalysts highly active for toluene complete oxidation** Catal Lett 2008 9 3–4 294 300

[B26] Mahendiran R Tiwary S Raychaudhuri A Ramakrishnan T Mahesh R Rangavittal N Rao C **Structure, electron-transport properties, and giant magnetoresistance of hole-doped LaMnO**_ **3** _** systems** Phys Rev B 1996 9 6 3348 3358 10.1103/PhysRevB.53.3348 9983844

[B27] Mizusaki J Yonemura Y Kamata H Ohyama K Mori N Takai H Tagawa H Dokiya M Naraya K Sasamoto T Inaba H Hashimoto T **Electronic conductivity, Seebeck coefficient, defect and electronic structure of nonstoichiometric La **_1***−******x***_**Sr**_ ** *x* ** _**MnO**_ **3** _Solid State Ion 2000 9 3–4 167 180

[B28] Shimura T Hayashi T Inaguma Y Itoh M **Magnetic and electrical properties of La(y)A(x)Mn(w)O(3) (A**** *=* ****Na, K, Rb, and Sr) with perovskite-type structure** J Solid State Chem 1996 9 2 250 263 10.1006/jssc.1996.0234

[B29] Huang XY Miyazaki Y Kajitani T **High temperature thermoelectric properties of Ca **_1***−******x***_**Bi**_ ** *x* ** _**Mn **_1***−******y***_**V**_ ** *y* ** _**O **_3***−δ***_** (0**** *≤* **** *x* **** *=* **** *y* **** *≤* ****0.08)** Sol State Commun 2008 9 3 132 136 10.1016/j.ssc.2007.10.012

[B30] Koc R Anderson H **Electrical conductivity and Seebeck coefficient of (La, Ca)(Cr, Co)O**_ **3** _J Mater Sci 1992 9 20 5477 5482 10.1007/BF00541609

[B31] Kuo J Anderson H Sparlin D **Oxidation reduction behavior of undoped and Sr-doped LaMno3: defect structure, electrical conductivity, and thermoelectric power** J Solid State Chem 1990 9 1 55 63 10.1016/0022-4596(90)90064-5

[B32] Ritter C Ibarra M DeTeresa J Algarabel P Marquina C Blasco J Garcia J Oseroff S Cheong S **Influence of oxygen content on the structural, magnetotransport, and magnetic properties of LaMnO3+delta** Phys Rev B 1997 9 14

[B33] Mizusaki J Mori N Takai H Yonemura Y Minamiue H Tagawa H Dokiya M Inaba H Naraya K Sasamoto T Hashimoto T **Oxygen nonstoichiometry and defect equilibrium in the perovskite-type oxides La **_1***−******x***_**Sr**_ ** *x* ** _**MnO**_ **3** _Solid State Ion 2000 9 3-4 163 177

[B34] Zeng Z Greenblatt M Croft M **Large magnetoresistance in antiferromagnetic CaMnO3-delta** Phys Rev B 1999 9 13 8784 8788 10.1103/PhysRevB.59.8784

[B35] Taylor PS Korugic-Karasz L Wilusz E Lahti PM Karasz FE **Thermoelectric studies of oligophenylenevinylene segmented block copolymers and their blends with MEH-PPV** Synth Met 2013 9 109 114

[B36] Shi H Liu C Xu J Song H Lu B Jiang F Zhou W Zhang G Jiang Q **Facile fabrication of PEDOT:PSS/polythiophenes bilayered nanofilms on pure organic electrodes and their thermoelectric performance** ACS Appl Mater Interfaces 2013 9 24 12811 12819 10.1021/am404183v 24313672

[B37] Yoon C Kim J Sung H Lee H **Electrical conductivity and thermopower of phosphoric acid doped polyaniline** Synth Met 1997 9 1–3 789 790

